# Behavioral Effects of a Novel Benzofuranyl-Piperazine Serotonin-2C Receptor Agonist Suggest a Potential Therapeutic Application in the Treatment of Obsessive–Compulsive Disorder

**DOI:** 10.3389/fpsyt.2017.00089

**Published:** 2017-05-22

**Authors:** Michelle M. Rodriguez, Carl Overshiner, J. David Leander, Xia Li, Denise Morrow, Richard G. Conway, David L. Nelson, Karin Briner, Jeffrey M. Witkin

**Affiliations:** ^1^Neuroscience Discovery Research and Discovery Chemistry, Lilly Research Laboratories, Eli Lilly and Company, Indianapolis, IN, USA

**Keywords:** obsessive–compulsive disorder, 5-HT_2C_ receptors, schedule-induced polydipsia, marble-burying, fluoxetine, Ro 60-0175, SB242084

## Abstract

Selective serotonin reuptake inhibitors (SSRIs) are the only effective pharmacological treatments for obsessive–compulsive disorder (OCD). Nonetheless, their generally limited efficacy, side-effects, and delayed onset of action require improved medications for this highly prevalent disorder. Preclinical and clinical findings have suggested serotonin2C (5-HT_2C_) receptors as a potential drug target. Data in rats and mice are presented here on the effects of a novel 5-HT_2C_ receptor agonist ((3S)-3-Methyl-1-[4-(trifluoromethyl)-7-benzofuranyl]-piperazine) (CPD 1) with high potency and full efficacy at 5-HT_2C_ receptors and less potency and partial agonism at 5-HT_2A_ and 5-HT_2B_ receptors. Effects of CPD 1 on consummatory (schedule-induced polydipsia in rats) and non-consummatory behaviors (marble-burying and nestlet-shredding in mice) that are repetitive and non-habituating were studied. We also evaluated the effects of CPD 1 in rats with isoproterenol- and deprivation-induced drinking in rats to compare with the polydipsia studies. The SSRIs, fluoxetine, and chlomipramine decreased the high rates of drinking in rats engendered by a schedule of intermittent food delivery (schedule-induced polydipsia). The effects of fluoxetine, but not of d-amphetamine, were prevented by the selective 5-HT_2C_ receptor antagonist SB242084. The 5-HT_2C_ receptor agonists Ro 60-0175 and CPD 1 also decreased drinking, but unlike the SSRIs and Ro 60-0175, CPD 1 dose-dependently decreased excessive drinking without affecting lever press responses that produced food. The effects of CPD 1 were prevented by SB242084. CPD 1 also suppressed drinking induced by isoproterenol and by water deprivation without affecting normative drinking behavior. CPD 1, like fluoxetine, also suppressed marble-burying and nestlet-shredding in mice at doses that did not affect rotarod performance or locomotor activity. The behavioral specificity of effects of CPD 1 against repetitive and excessive behaviors suggests a potential therapeutic application in OCD.

## Introduction

Obsessive–compulsive disorder (OCD) was formally classified as an anxiety disorder (DSMIV) but was recently given its own category (DSMV) due to greater commonalities found between this disorder and other OCD-related disorders. The prevalence of the disorder is quite high with estimates of 1–3% of the adult population and greater in children (1–3). OCD and OCD symptoms also demonstrate high comorbidity with other psychiatric disorders (4, 5). In contrast to generalized anxiety disorders that can be fairly well managed by benzodiazepines or selective serotonin reuptake inhibitors (SSRIs), patients with OCD are poorly served by current psychotherapic and pharmacotherapeutic interventions (2, 6), and it is reported that >10% of patients do not respond to any of the available treatments (7). SSRIs (e.g., chlomipramine, fluoxetine) are the only effective pharmacotherapies for OCD, and yet their efficacy is limited. SSRIs require doses that are generally well in excess of the doses required for treatment of depression or anxiety. The higher doses used increase the risk of side effects and compliance becomes a medical management issue. In addition, the therapeutic onset for OCD treatment is generally much greater than the several weeks required for antidepressant efficacy, with treatment periods sometimes lasting many months before symptomatic relief is achieved. A further failing of the SSRIs used for treatment of OCD is that even when they demonstrate symptom suppression in an individual, the overall symptom improvement is not typically impressive (2). Thus, there is room for substantial improvement in the pharmacological management of OCD.

As the only effective pharmacological therapy for OCD, the mechanism of action of SSRIs can be used as a starting point for speculations about potential methods for improving drug treatments. By selectively inhibiting the neuronal uptake of serotonin, SSRIs increase overall synaptic availability of serotonin, the postsynaptic translations of which are dependent upon serotonin binding. There are currently fourteen such serotonin receptors classified by ligand binding and pharmacological response into seven subtypes (5HT_1–7_) (8). The actions of serotonin at any one or combination of these postsynaptic receptors could be responsible for the therapeutic benefit of SSRIs in OCD. There are several pieces of information that have implicated 5-HT_2C_ receptors in this process.

Although the molecule has ancillary actions, *m*-chlorophenylpiperazine has high affinity for 5-HT_2__C_ receptors. A handful of clinical studies have been conducted with mCPP that also suggest the potential viability of 5-HT_2__C_ receptor agonism as a mechanism for OCD therapeutics. When given acutely, mCPP blunted neuroendocrine responses in OCD patients, whereas dopamine and norepinephrine responses were not different from non-OCD patients (2). mCPP has also been reported to increase OCD symptomatology, an effect not generally produced by anxiogenic agents; this effect of mCPP is recapitulated in the first few days of dosing with SSRI and OCD therapeutic agent, clomipramine, in OCD patients [Zohar, 1987, unpublished data, (9)]. Exacerbation of OCD symptoms after acute challenge with mCPP are completely prevented by the 5-HT_1_/5-HT_2_ antagonist, metergoline (10). Another piece of clinical data of relevance is the observation that the augmentation in OCD symptomotology is absent in patients who have undergone chronic dosing with clomipramine or fluoxetine (9, 11). Finally, limited data have been disclosed on the effects of chronic administration of mCPP. In one study, significant reductions in OCD symptoms were reported with clomipramine showing greater efficacy than mCPP (12). The clinical data with mCPP suggesting a role for 5-HT_2C_ receptors in OCD are supported by observations of mice with deletions of the 5-HT_2C_ receptor. These mice demonstrate perseverative head-dipping and a pattern of gnawing that was interpreted as compulsive when the receptor knockout mice were compared to their wild-type controls (13).

More definitive investigations in the potential roles of 5-HT_2C_ receptors in the control of OCD will be greatly aided by the use of pharmacological-specific tools. CPD 1 is a novel benzofuranylpiperazine molecule ((3S)-3-methyl-1-[4-(trifluoromethyl)-7-benzofuranyl]-piperazine). CPD 1 has high affinity and selectivity for 5-HT_2C_ receptors as described here (Table 1). The present study was conceived and executed in order to characterize some behavioral effects of CPD 1 in terms of its potential as an OCD treatment candidate. Comparisons in some cases were made with SSRIs, as well as other 5-HT_2C_ receptor agonists. The overall findings that CPD 1 suppressed a host of excessive and repetitive behaviors without notable effects on ancillary behaviors suggest that 5-HT_2C_ receptor agonists may be efficacious in the clinical management of OCD.

**Table 1:** **Affinity was measured using standard radioligand binding techniques employing agonist radioligands**.

Receptor	Mean (nM)	SEM	*N*
h5-HT1A	205	39.9	6
h5-HT1B	1,115	479	8
h5-HT1D	296	55.5	6
h5-HT1E	1,035	180	7
h5-HT1F	540	173	6
h5-HT_2A_	47.2	9.28	6
h5-HT_2B_	104	26.2	6
h5-HT_2C_	2.11	0.47	6
h5-HT_4_	977	354	2
h5-HT_6_	212	29	6
h5-HT_7_	>2 μM	–	4

*Each value is given as the mean ± SEM for the number of separate experiments given in parentheses, with the following exceptions: for the 5–HT_4_ receptor, the value is the mean ± one-half the range of the mean. For the 5–HT_7_ receptor, less than 50% inhibition of binding was seen at a concentration of 2 µM CPD1*.

## Materials and Methods

### Receptor Binding

Binding affinities at the serotonin receptors were determined by standard radioligand-binding methods using human cloned receptors as reported in the literature (14, 15).

### GTP-γ-[^35^S] Methods

5-HT_2_ receptors are functionally coupled to specific G-proteins. Agonist activation of 5-HT_2_ G-protein coupled receptors results in the release of GDP from alpha-subunit (G alpha q or G alpha i) of the G-protein and the subsequent binding of GTP. The binding of the stable analog GTP-g-[^35^S] is an indicator of receptor activation. Human cloned 5-HT_2A_, _−2B_, and _−2C_ receptors were expressed in AV12 cells. GTP-g-[^35^S] nucleotide exchange scintillation proximity assay (SPA) was used to measure binding as previously described (14).

### Broad Receptor Screening Panel

Additional selectivity assessment of CPD 1 was performed at CEREP. Methods are available for the multiple assays utilized at http://www.cerep.fr/cerep/users/pages/catalog/assay/catalog.asp. The specific ligand binding to the receptors is defined as the difference between the total binding and the non-specific binding determined in the presence of an excess of unlabeled ligand. The results are expressed as a percent inhibition of control specific binding obtained in the presence of CPD 1 evaluated at 10 µM in duplicate.

### Behavioral Pharmacology

#### Animals

The facilities in which the animals were maintained are fully accredited by the American Association for the Accreditation of Laboratory Animal Care (AAALAC), and the studies described herein were conducted in accordance with the Guide for Care and Use of Laboratory Animals under protocols approved by a local animal care and use committee. All work was done in accordance with U.S. Public Service Policy on Humane Care and Use of Laboratory Animals, amended August 2002. Furthermore, all research protocols were approved by an internal Animal Care and Use Committee.

Male, NIH Swiss mice (Harlan Labs, Indianapolis, IN, USA) weighed between 28 and 32 g and were housed in groups of 10–12 in plastic cages (24 cm × 45 cm × 15 cm high) with sawdust bedding in a temperature-controlled vivarium. A 12-h light/dark cycle was maintained, and all experimental sessions were conducted during the light phase of the cycle. The sessions were conducted between 8:00 and 10:00 a.m. daily.

#### Schedule-Induced Polydipsia

Rats were used in these experiments as they have been utilized for such studies in the past with great success in generating significant polydipsia (16). Male, Wistar rats were singly housed, and maintained at approximately 85% of their free-feeding weights with free access to water. Twelve adult male, Wistar rats (Harlan Industries, Indianapolis, IN, USA) were used. They were allowed to acclimate to our vivarium for at least 3 days prior to testing. They were 2 months old at the start of the experiments that lasted approximately 6–8 months and were without explicit experimental experience prior to this study. The rats were singly housed, and maintained at approximately 86–91% of their free-feeding weights with post session supplemental feeding of Lab Diet #5001 for rodents (PMI Nutrition International Inc., St. Louis, MO, USA). Body weights were corrected for the normal growth curve of Wistar rats such that actual body weight of each rat increased over the experimental time frame. Water was available continuously in the individual home cages. Rats were inspected daily 7 days/week by experimenters and veterinary staff. Under the conditions of this experiment, there were no health issues observed.

The experiments were conducted using operant behavior test chambers ENV-007 (Med Associates Inc., Georgia, VT, USA), 30.5 cm × 24.1 cm × 29.2 cm. The test chambers were contained within light and sound attenuating shells. A food trough was mounted 2 cm off the grid floor on the centerline. Two response levers were centered 8 cm off the centerline and 7 cm off the grid floor. Three lights were located above each response lever at 15 cm off the grid floor. All events were controlled and lever-press data were recorded by a Compaq computer running MED-PC Version IV (Med Associates Inc., Georgia, VT, USA). The water bottle located within each chamber was weighed before and after experimental sessions.

Rats responded under a fixed-interval 120 s schedule of food delivery in which the first lever press to occur after the elapse of 120 s resulted in the delivery of a 45 mg food pellet. Experimental sessions lasted 90 min. Test compounds or drug vehicle were administered on Tuesdays and Fridays, given the stability of performance (within 15% variation of the individual animal). Non-injection, control performances were on Thursdays. Groups of five to eight rats were randomly selected from the pool of animals that demonstrated stable performance and given one dose of vehicle or test compound. Dose-effect curves and drug interaction data were created by repeated testing in groups of five to eight rats. Test compounds were administered as described in figure legends. The effects of the compound are expressed as a percent of control for each individual animal. Results were analyzed by ANOVA followed by Dunnett’s multiple comparison (*p* < 0.05).

Although the number of animals/dose were relatively small (5–8/dose group), the effect size was large and the control behavior was tightly controlled and, therefore, sufficient for detection of statistically significant drug effects. Thus, in keeping with the guidelines of animal care and use followed here, we utilized the minimum number of animals required for signal detection. Although order effects from experiment to experiment cannot be ruled out in the present design, the behavior of all animals returned to baseline control values for at least 3 days prior to the animal being used in another drug test. In some cases, systematic replication (e.g., same dose of fluoxetine given in another experiment) was used to verify that drug effects were qualitatively consistent across replication.

#### Deprivation-Induced Drinking

Male, Wistar rats (different from those rats used in the schedule-induced polydipsia studies) were used. They were 320 ± 15 g at the initiation of this study. Water was removed from their home cage for 18 h after which water bottles were presented to each rat in an individual housing cage in a quiet room for 60 min. The weight of the water bottle before and after the 60 min period was measured to the nearest 0.1 g. Six to eight rats were studied per dose. It is noted that this deprivation period is considered stressful. After the experiment, the animals were returned to their living cages with continuous access to food and water. No health effects were observed in these animals by direct visual observation and handling.

#### Isoproterenol-Induced Drinking

Male, Wistar rats (different from those rats used in the schedule-induced polydipsia studies) were used. They were 331 ± 11 g at the initiation of this study. Compound 1 (5.6 mg/kg, s.c.) was evaluated for its ability to reduce drinking. (±)-isoproterenol (30 μg/kg, s.c.) was given to induce drinking (*n* = 5–20 rat/group). Ten minutes after vehicle or (±)-isoproterenol injection, rats were given either vehicle or Compound 1 and a 60 min session began in which the rats in individual housing cages were given access to water 20 min after the second injection. The weight of the water bottle before and after the 60 min period was measured to the nearest 0.1 g. After the experiment, the animals were returned to their living cages with continuous access to food and water. No health effects were observed in these animals by direct visual observation and handling.

#### Nestlet-Shredding

Mice (8/dose group) were used in these studies as the behavior has been well characterized in this species and strain as described here. Studies with mice were conducted as described (17). Mice were acclimated to the experimental room for 60 min under normal overhead fluorescent lighting. Mice were subsequently dosed with vehicle or compound and after a specified pretreatment interval (generally 30 min), were placed in a 17 cm × 28 cm × 12 cm high plastic tub with ~ 5 mm sawdust shavings on the floor along with a preweighed multiply gauze pad (51 mm^2^; 3 g). Mice were left in the tub for 60 min after which the weight of the gauze pad that had not been torn off by the mouse was obtained. The weight of the gauze used for nest construction was determined by subtraction.

#### Marble Burying and Rotarod

Studies with mice (12/dose group) were conducted as described (17) since this behavior is robust in this species and mouse strain. Separate groups of mice were used in these experiments that were conducted in a dimly lit testing room. The mice were not injected with any drug or vehicle more than once in these studies as the same mouse was evaluated post drug administration sequentially on the rotarod task and then in the marble-burying assay. After 60 min acclimation to the experimental room, mice were dosed with vehicle or compound and after a specified pretreatment interval (noted in the drug section immediately below), were placed on a rotarod (Ugo Basile 7650) operating at a speed of 6 revolutions/min, and observed for falling. Mice that fell off the rotarod on two occasions during 2 min were scored as failing. Mice were not pretrained on this task. After the rotarod task, they were placed in a 17 cm × 28 cm × 12 cm high plastic tub with 5 mm sawdust shavings on the floor that were covered with 20 blue marbles (1.5 cm diameter) placed in the center. Mice were left in the tub for 30 min. The number of marbles buried (2/3 covered with sawdust) was counted.

#### Locomotor Activity

Locomotor activity of mice (12/dose group) was assessed in order to compare the drug effects thereon to the effects in this species in marble-burying and nestlet shredding. Locomotor activity was measured with a 20 station photobeam activity system (San Diego Instruments, San Diego, CA, USA) with seven photocells per station. Locomotor activity was recorded as the number of ambulations, where ambulation was defined as the breaking of adjacent photobeams. Separate groups of mice were tested. Mice were injected with either vehicle or compound and placed into a plastic tub measuring 41 cm × 20 cm × 15 cm for 60 min. Seven photobeam emittors and detectors were spaced 6 cm apart along the length of the cage. Successive breaks of photobeams (locomotion) were counted in 10 min intervals.

#### Data Analysis

Effects of compounds on schedule-induced polydipsia, marble burying, nestlet shredding, and locomotor activity were analyzed by ANOVA followed by *post hoc* Dunnett’s tests, and the effects on rotarod performance were assessed by Fisher’s exact test. The smallest dose required to significantly produce an effect was defined as the minimal effective dose (MED). ED_50_ values (±95% confidence limits) were calculated from log-linear regression analysis of the dose–response curves. In studies with two variables (e.g., day and treatment), two-way ANOVA was used. Statistical probabilities of less than 0.05 were considered to be significant.

#### Drugs

Chlordiazepoxide HCl, pentobarbital Na, d-amphetamine SO_4_, chlorpromazine HCl, clomipramine HCl, and isoproterenol HCl (Sigma Chemical Co., St. Louis, MO, USA), MDL100907 (Tocris Bioscience, Bristol, UK), and citalopram HBr (synthesized by Eli Lilly and Co., Indianapolis IN, USA). were dissolved in 0.9% NaCl. All compounds were prepared just prior to dosing and administered i.p. in a volume of 1 ml/kg body weight (mice) or 10 ml/kg body weight (rats). Fluoxetine was administered s.c. For marble burying, compounds were administered either 30 min, 15 min (pentobarbital), or 10 min (d-amphetamine) prior to behavioral testing. For nestlet shredding and locomotor acitivity, compounds were administered immediately prior to testing. Drug doses are expressed as the salt.

## Results

### Pharmacological Characterization of CPD1

CPD 1 was shown to have high affinity for 5-HT_2C_ receptors (Table 1) and to demonstrate selectivity for 5-HT_2C_ receptors over the other serotonin receptor subtypes (Table 1). Specifically, CPD 1 is 22-fold selective over h5-HT_2A_ receptors, 50-fold selective over h5-HT_2B_ receptors, and at least 100-fold selective over other cloned h5-HT receptors.

**Figure 1:**
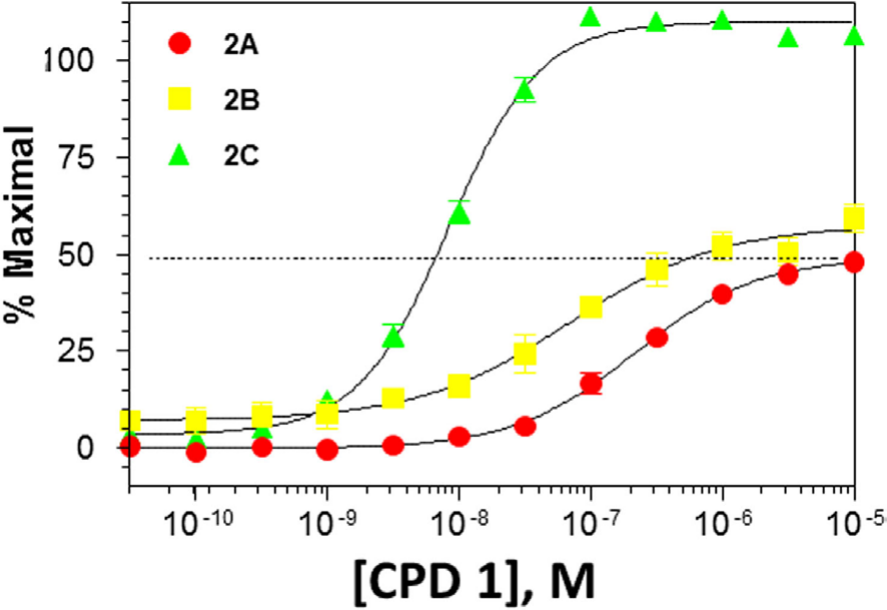
**Functional activity of CPD1 at 5-HT_2A,B,C_ receptors**. Data are from experiments conducted with *n* = 15. Each data point represents the mean ± SEM.

Functional activity of CPD 1 at 5HT_2_ receptors showed that this molecule is a full agonist at 5-HT_2C_ receptors with high potency. In contrast, CPD 1 functions as a partial agonist at 5-HT_2A_ and 5-HT_2B_ receptors with lower affinities than for 5-HT_2C_ receptors (Figure 1; Table 2).

**Table 2:** **Potencies and efficacies of three 5-HT_2C_ receptor agonists in a GTPgs functional assay using human recombinant cell lines**.

Compound	Structure	H5-HT_2A_	h5-HT_2B_	h5-HT_2C_
CPD 1	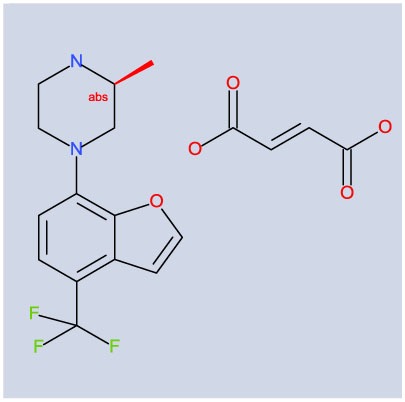	269 ± 19 (28)	97 ± 23 (27)	8.1 ± 0.5 (31)
		53 ± 2 (29)	51 + 19 (27)	110 ± 1 (31)

Ro 60-0175	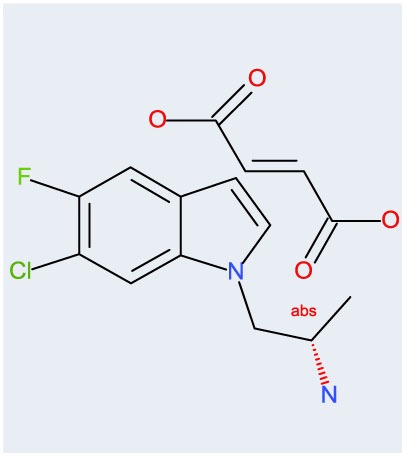	130 ± 1 (118)	57 ± 1 (123)	5.1 ± 1 (123)
		59 ± 1 (124)	58 ± 1 (105)	104 ± 1 (123)

mCPP	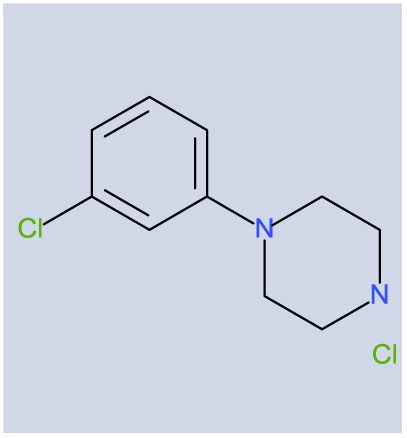	97 ± 1 (9)	2 ± 1 (8)	1 ± 0 (7)
		88 ± 2 (9)	84 ± 2 (8)	99 ± 2 (7)

*Values are means ± SEM (*n*). The top value for each compound is the EC_50_ in nanomoles. The bottom value for each compound is the Emax in %*.

CPD 1 was also evaluated for its affinity to a broad array of other protein targets. Of the 68 proteins (receptors, transporters, enzymes) tested with CPD 1 at a concentration of 10 µM, 16 of these (23.5%) bound CPD 1 sufficiently that >50% inhibition of binding was observed (Table 3).

**Table 3:** **Proteins in which CPD 1 (10 mM) displaced radioligand by >50%**.

Protein	α1	α2	β2	D1	D2s	D3	D4.4	D5	H1	H2	M1	M3	L-Verapamil site	Na+ Channel	NE transporter	σ
% Inhibition	82	70	104	94	61	82	105	69	93	70	56	76	59	101	74	100

### Effects of SSRIs on Schedule-Induced Polydipsia

Control, non-drug baseline levels of lever pressing and water consumption under the fixed-interval schedule were 1,005 + 20.1 responses and 22.3 + 1.15 ml water consumed across the 21 control sessions conducted. The variation across these sessions was <10% with the exception of two sessions in which response variation was 13% and water consumption variation from mean was 15% on one experimental session. Behavior generally returned to baseline values the day after drug administration or 2 days later if the animals were dosed on a Friday (no experimental sessions were run on weekends).

**Figure 2:**
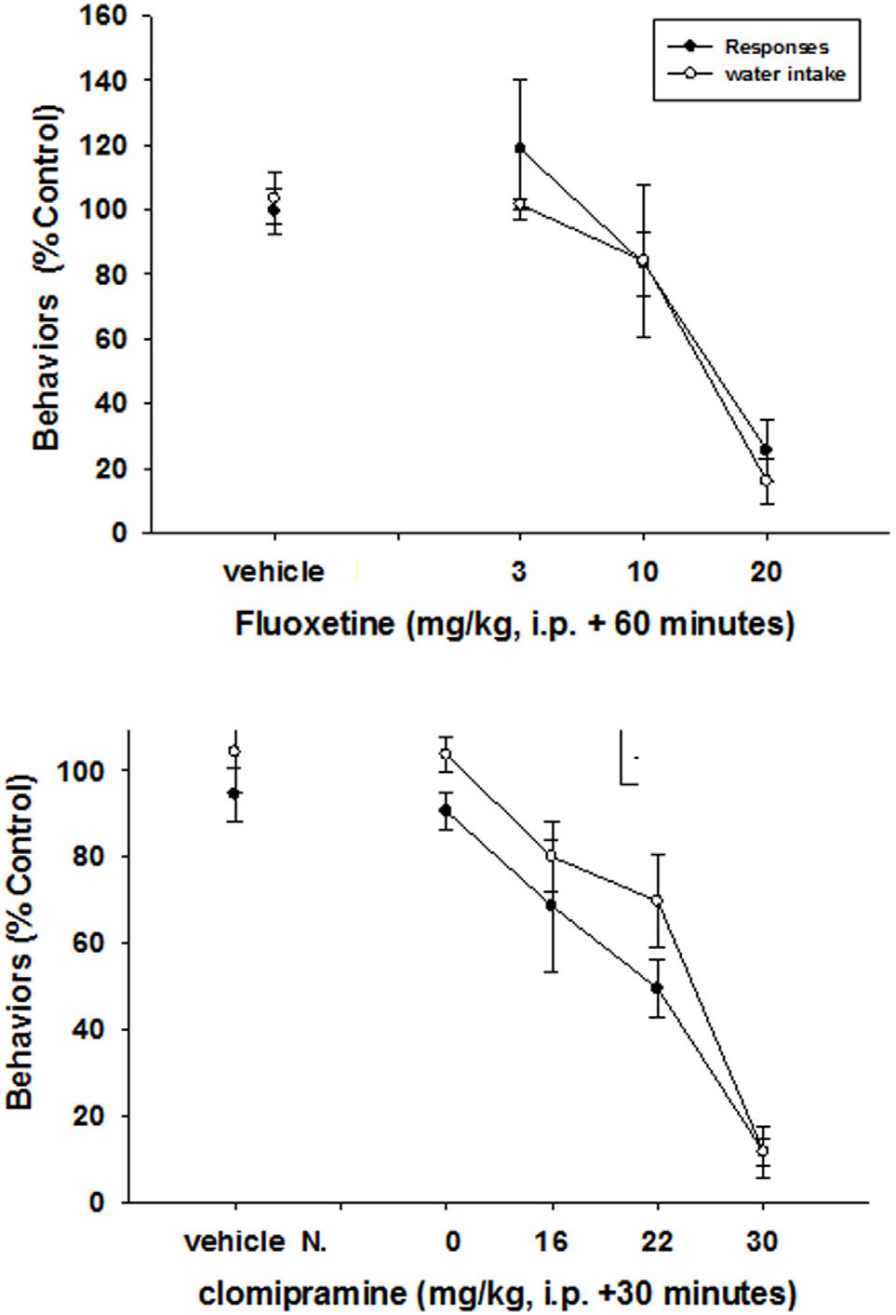
**Effects of SSRIs on schedule-induced polydipsia**. Significant effects on the number of responses and amount of water consumed were produced by both fluoxetine (*F*_2,23_ = 4.4, *p* < 0.001—responses; *F*_2,23_ = 25, *p* < 0.0001—water) and clomipriamine (*F*_3,31_ = 12.6, *p* < 0.0001—responses; *F*_3,31_ = 20.5, *p* < 0.0001). Each point represents the mean ± SEM of eight rats.

Fluoxetine (3–20 mg/kg) produced a general reduction in both food-maintained responding and excessive drinking. Statistically significant reductions of both food-maintained responding (*F*_2,23_ = 4.4, *p* < 0.001) and excessive drinking (*F*_2,23_ = 25, *p* < 0.0001) were detected at a dose of 20 mg/kg of fluoxetine (Figure 2). Clomipramine also decreased both responses (*F*_3,31_ = 12.6, *p* < 0.0001) and excessive drinking (*F*_3,31_ = 20.5, *p* < 0.0001) at generally comparable doses (Figure 2).

### Prevention of Effects of Fluoxetine by a 5HT_2C_ Receptor Antagonist

**Figure 3:**
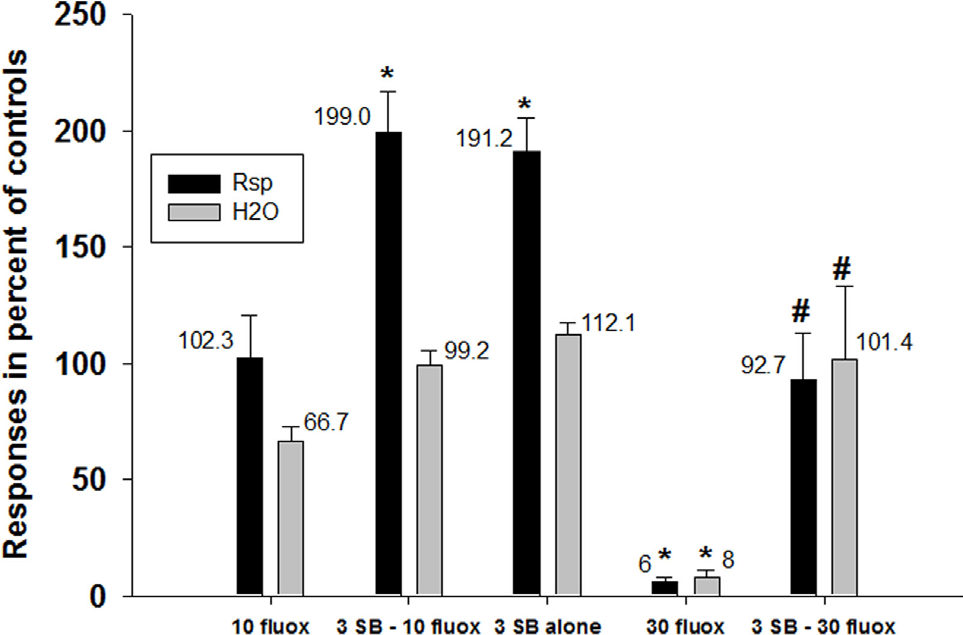
**Prevention of effects of fluoxetine by a 5HT_2C_ receptor antagonist**. Fluoxetine (30 mg/kg, i.p.) significantly suppressed both the number of responses emitted and the amount of water consumed in rats under a schedule of food delivery engendering excessive drinking. Co-dosing with SB-242084 (3 mg/kg) significantly attenuated these decreases. Each bar represents the mean ± SEM of five to eight rats. *p < 0.05 compared to vehicle (Dunnett’s test). ^#^*p* < 0.05 compared to fluoxetine alone (30 mg/kg) (Dunnett’s test).

The 5HT_2C_ receptor antagonist SB242084 (18) significantly attenuated the decreases in water consumption and response output under the schedule-induced polydipsia procedure produced by fluoxetine (Figure 3). In this figure, the effects of 10 mg/kg fluoxetine are shown to have no significant effect when given alone (first set of two bars). In conjunction with SB242084, rates of responding are significantly elevated (second set of two bars) as they are with SB242084 when given alone (third set of two bars). A half-log higher dose of fluoxetine (30 mg/kg) markedly suppressed both responding and water consumption (fourth set of two bars), and this effect was blocked SB243084 (last set of two bars).

**Figure 4:**
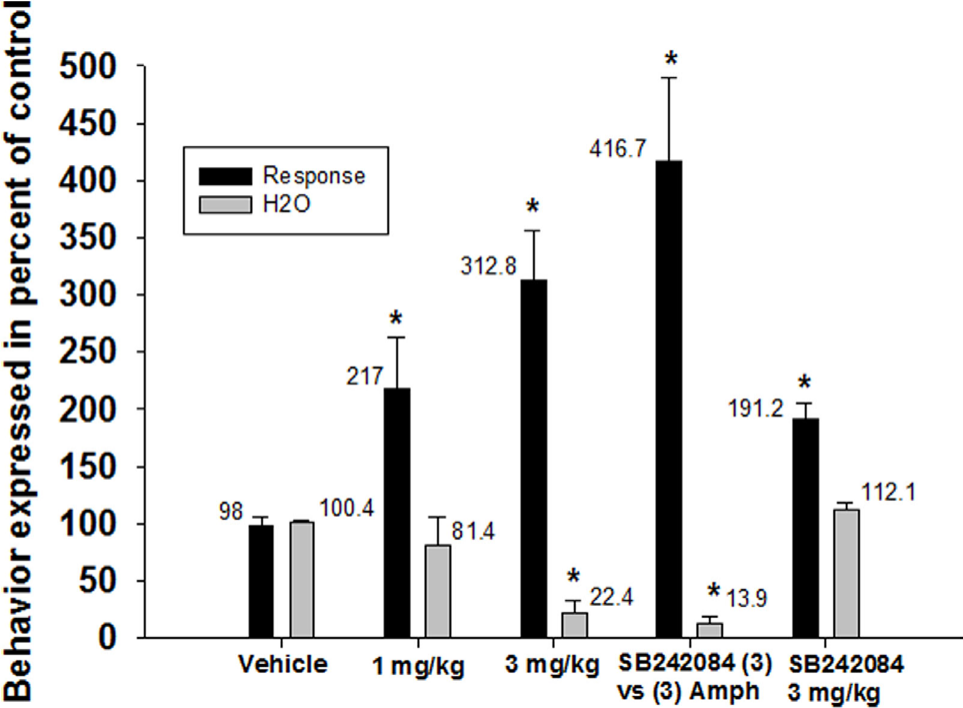
**Effects of 5-HT_2C_ receptor antagonism on the reductions in schedule-induced polydipsia produced by d-amphetamine**. d-Amphetamine significantly enhanced the number of responses emitted (*F*_2,19_ = 18.4, *p* < 0.0001) and significantly decreased the amount of water consumed (*F*_2,19_ = 24.9, *p* < 0.0001). SB-242084 did not block these effects of d-amphetamine Each bar represents the mean ± SEM of five to eight rats. *p < 0.05 compared to vehicle (Dunnett’s test).

In contrast, SB242084 did not alter the effects of d-amphetamine on schedule-induced polydipsia (Figure 4). Given alone, d-amphetamine increased responding (*F*_2,19_ = 18.4, *p* < 0.0001) and decreased the amount of water consumed (*F*_2,19_ = 24.9, *p* < 0.0001). SB242084 when given alone increased responding but did not significantly affect water consumption.

#### Effects of 5HT_2C_ Receptor Agonists on Schedule-Induced Polydipsia

**Figure 5:**
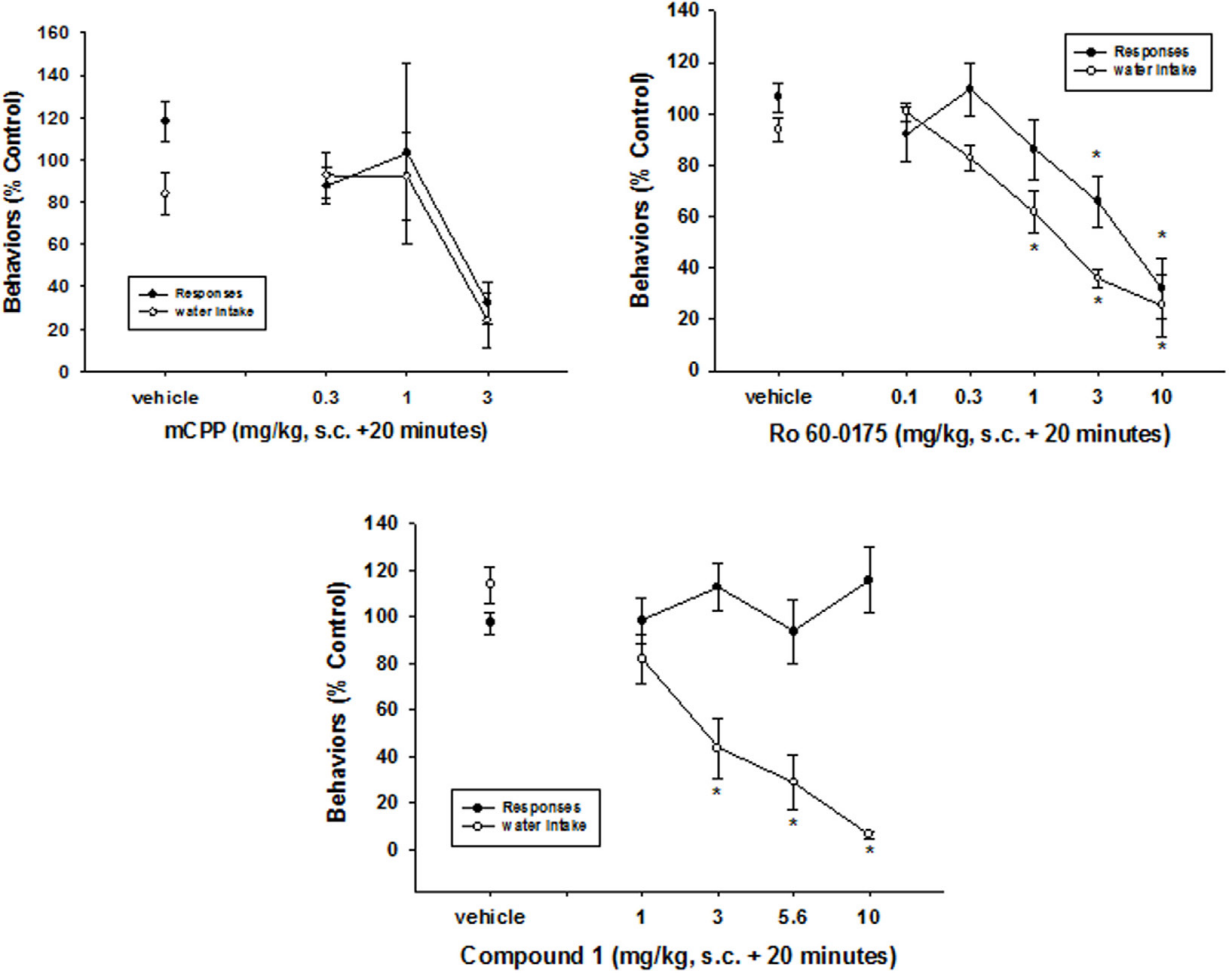
**Effects of 5HT_2C_ receptor agonists on schedule-induced polydipsia**. mCPP and Ro 60-0175 significantly decreased both the number of responses emitted and the amount of water consumed (mCPP: reponses—*F*_2,23_ = 17.1, *p* < 0.0001; water—*F*_2,23_ = 13.5, *p* < 0.001) (Ro-60-0175: responses—*F*_4,39_ = 8.6, *p* < 0.0001; water—*F*_2,23_ = 13.5, *p* < 0.001). In contrast, CPD1 dose-dependently suppressed excessive water drinking (*F*_3,31_ = 9.5, *p* < 0.001) without affecting response output (*F*_3,31_ = 0.37, *p* = 0.78). Each point represents the mean ± SEM of five to eight rats. *p < 0.05 compared to vehicle (Dunnett’s test).

The 5-HT_2C_ receptor agonists, mCPP (PDSP Data Base; Table 2) and Ro 60-0175 [(19, 20); Table 2], like fluoxetine, significantly decreased both the number of responses emitted and the amount of water consumed (mCPP: responses—*F*_2,23_ = 17.1, *p* < 0.0001; water—*F*_2,23_ = 13.5, *p* < 0.001) (Ro-60-0175: responses—*F*_4, 39_ = 8.6, *p* < 0.0001; water—*F*_2,23_ = 13.5, *p* < 0.001) (Figure 5). In contrast, CPD1 dose-dependently suppressed excessive water drinking (*F*_3,31_ = 9.5, *p* < 0.001) without affecting response output (*F*_3,31_ = 0.37, *p* = 0.78). Statistically significant reductions of excessive drinking were detected at doses of 3 mg/kg and greater of CPD 1 (Figure 5).

#### Effects of 5-HT_2C_ Receptor Antagonism on the Reductions in Schedule-Induced Polydipsia

**Figure 6:**
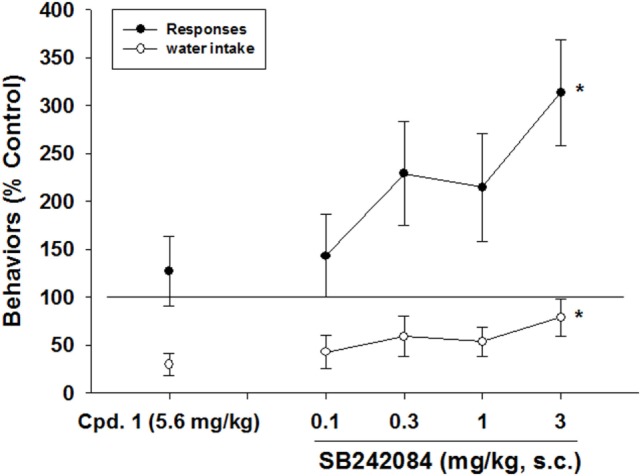
**Effects of 5-HT_2C_ receptor antagonism on the reductions in schedule-induced polydipsia produced by CPD 1**. SB-242084prevented the decreases in excessive water consumption produced by CPD 1 (5.6 mg/kg) (*F*_4,25_ = 6.0, *p* < 0.001) and significantly enhanced the number of responses emitted (*F*_4,25_ = 3.1, *p* < 0.05). Each point represents the mean ± SEM of 6 rats/group. *p < 0.05 compared to vehicle (Dunnett’s test).

SB-242084 prevented the decreases in excessive water consumption produced by CPD 1 (5.6 mg/kg) (*F*_4,25_ = 6.0, *p* < 0.001) and significantly enhanced the number of responses emitted (*F*_4,25_ = 3.1, *p* < 0.05) (Figure 6).

#### Effects of 5HT_2A_ Receptor Antagonism on the Reductions in Schedule-Induced Polydipsia

The 5-HT_2A_ receptor antagonist, MDL100907 (21) was used to access the potential contributions of 5-HT_2A_ receptors on the actions of CPD 1. Excessive water consumption was significantly reduced (64% decrease) by CPD 1 (5.6 mg/kg, sc, 20 min prior). MDL100907 (0.1 mg/kg, sc, 30 min prior) did not significantly alter the decrease produced by CPD 1 (65%, *p* > 0.05).

#### Effects of CPD 1 on Deprivation-Induced Drinking

**Figure 7:**
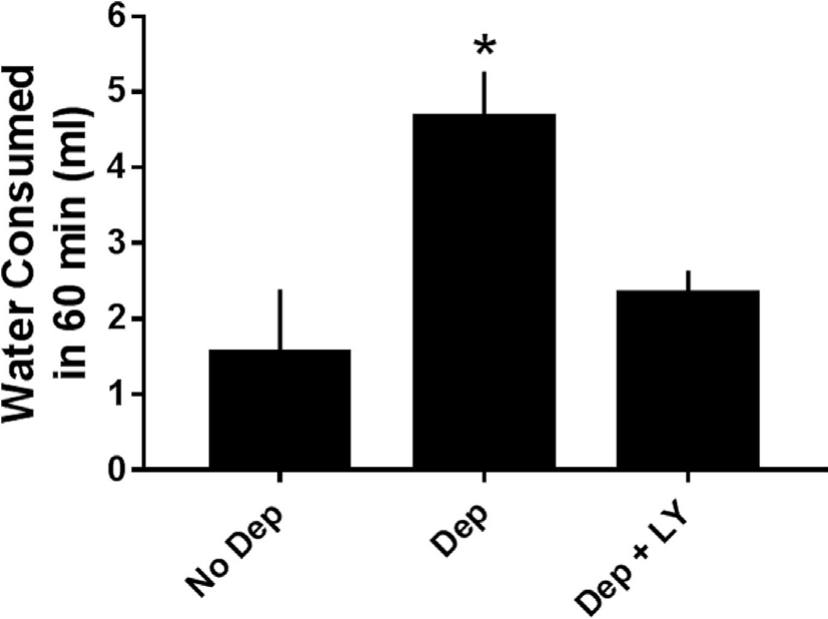
**Effects of CPD1 on deprivation-induced drinking**. Water deprivation significantly increased the amount of water consumed and this enhancement was prevented by CPD 1 (*F*_2,18_ = 7.5, *p* < 0.01). Data are means ± SEM of 6–8 rats/group. *p < 0.05 compared to vehicle (Dunnett’s test).

Water deprivation for 18 h engendered increases in water consumption. Deprivation-induced drinking was significantly reduced by 50% by 5.6 mg/kg of CPD 1 (Figure 7).

#### Effects of CPD 1 on Isoproterenol-Induced Drinking

**Figure 8:**
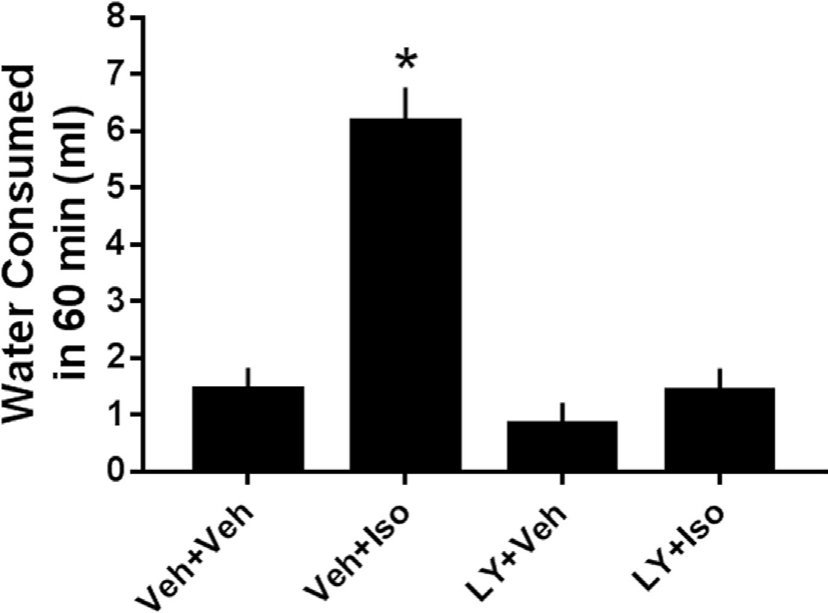
**Effects of CPD1 on isoproterenol-induced drinking**. Isoproterenol significantly increased the amount of water consumed and this enhancement was prevented by 5.6 mg/kg CPD 1 (*F*_3,48_ = 24.9, *p* < 0.0001) that did not significantly affect normal drinking (LY + Veh). Data are means ± SEM of 5 (vehicle)-20 rats/group. *p < 0.05 compared to vehicle (Dunnett’s test).

Isoproterenol increased water consumption by 432% relative to vehicle-treated rats. Isoproterenol-induced drinking was reduced by 77% by 5.6 mg/kg of CPD 1 (*F*_3,48_ = 24.9, *p* < 0.0001) (Figure 8). In contrast, CPD 1 did not significantly affect normal drinking (Figure 8).

#### Effects of 5-HT_2C_ Receptor Agonists on Marble-Burying and Nestlet-Shredding

Ro 60-0175 decreased marble-burying at a MED of 0.1 mg/kg and nestlet-shredding at a MED of less than 1 mg/kg. CPD 1 decreased marble-burying at a MED of 10 mg/kg and nestlet-shredding at a MED of 3 mg/kg (Table 4).

**Table 4:** **Effects of the 5-HT_2C_ agonists CPD 1 and Ro 60-0175 compared fluoxetine and pentobarbital on marble-burying, nestlet shredding, rotarod performance, and locomotor activity of mice**.

Compound	Marble-burying	Nestlet-shredding	Rotarod	Locomotor activity
Fluoxetine	10	10	>30	>10
CPD 1	10	3	>10	>10
CPD 1 + SB242084	>10	n.t.	>10	n.t.
RO 60-0175	0.1	<1	>10	>1
Pentobarbital	>17	10	5.6	n.t.

*Values are minimal effective doses in milligrams per kilogram. The number of mice used/dose group were 12 (marble-burying), 8 (nestlet shredding), 12 (rotarod), and 12 for locomotor activity*.

*n.t., not tested*.

#### Effects of 5HT_2C_ Receptor Agonists on Locomotor Activity and Rotarod Performances

Ro 60-0175 decreased locomotor activity at a MED of greater than 10 mg/kg and rotarod performances at a MED of greater than 1 mg/kg. CPD 1 decreased locomotor activity and rotarod performances at a MED of greater than 10 mg/kg (Table 4).

#### Effects of Repeated Dosing of 5-HT_2C_ Receptor Agonists on Behavior

**Figure 9:**
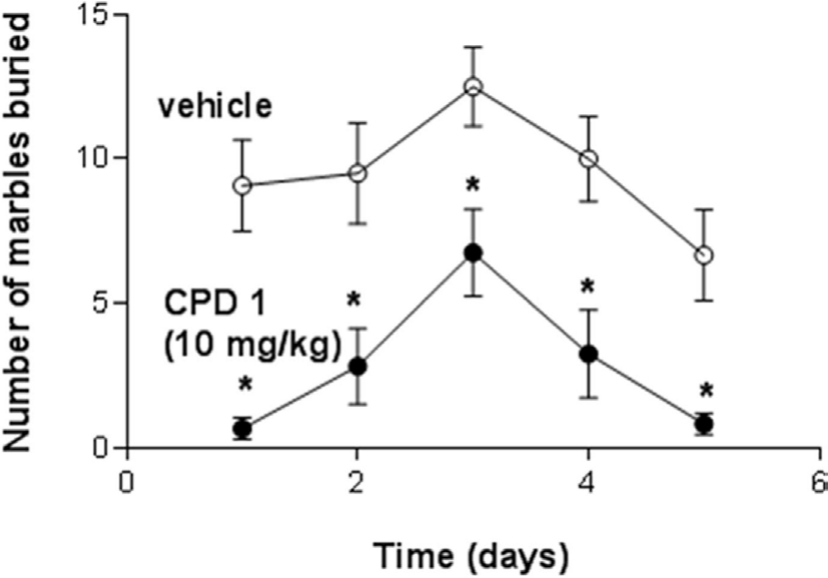
**Effects if CPD1 on marble-burying behavior of mice over five consecutive days**. Marble-burying was significantly decreased by CPD 1 (10 mg/kg) compared to vehicle-treated mice (*F*_1,110_ = 59.4, *p* < 0.0001). There was also a significant impact of dosing day (*F*_4,110_ = 5.2, *p* < 0.001) but no significant day × treatment interaction (*F*_4,110_ = 0.31, *p* = 0.87). Data are means ± SEM of 12 mice/group. *p < 0.05 compared to day 1 (Dunnett’s test).

Marble-burying was significantly decreased by CPD 1 (10 mg/kg) compared to vehicle-treated mice (*F*_1,110_ = 59.4, *p* < 0.0001). Repeated dosing with CPD 1 did not produce tolerance to its suppressant effects. Over 5 days of consecutive dosing effects of CPD 1 (10 mg/kg) on marble-burying did not significantly tolerate over days (Figure 9). There was also a significant impact of dosing day (*F*_4,110_ = 5.2, *p* < 0.001) but no significant day × treatment interaction (*F*_4,110_ = 0.31, *p* = 0.87).

**Figure 10:**
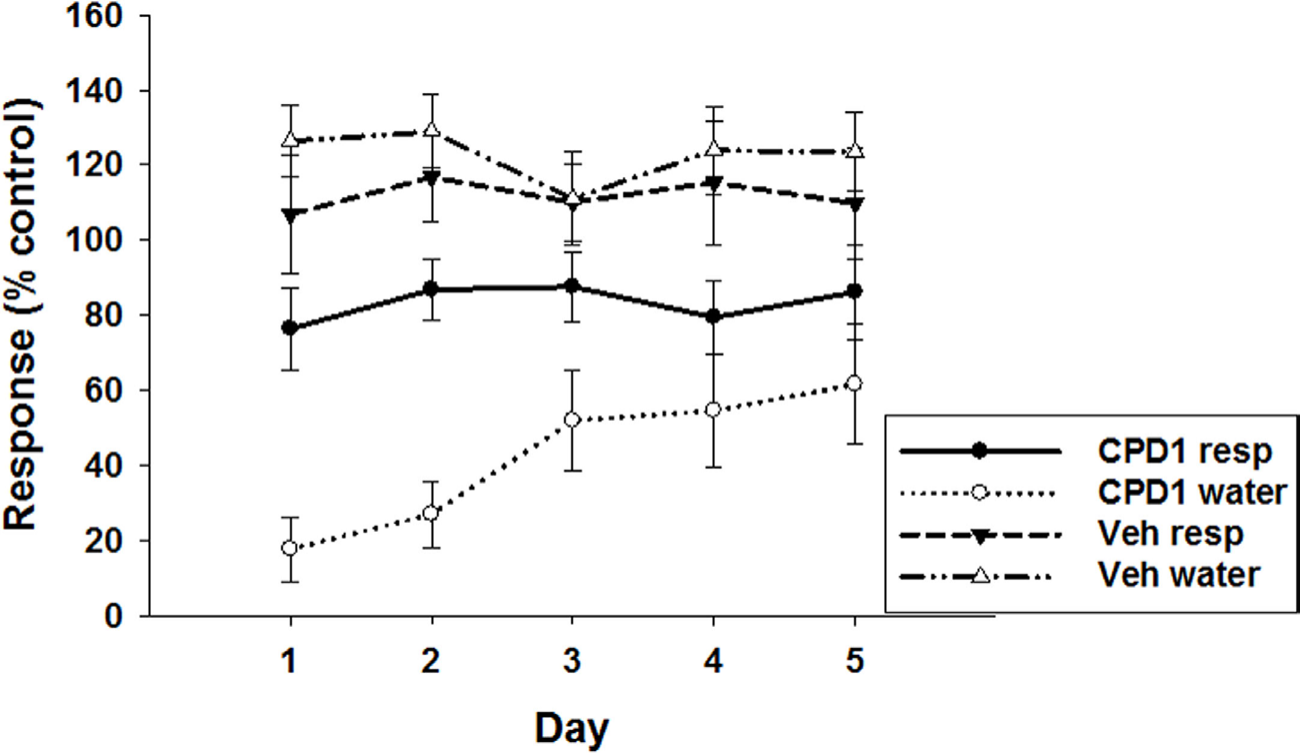
**Effects of CPD1 on schedule-induced polydipsia over five consecutive days of dosing**. CPD 1 (10 mg/kg) significantly decreased excessive water consumption (*F*_1,40_ = 128.5, *p* < 0.0001) and to a lesser extent response output over 5 days (*F*_1,40_ = 11.7, *p* < 0.05). The factor of day of dosing did not significantly impact effects of CPD1 (responses: *F*_4,40_ = 0.11, *p* = 0.98; water: *F*_4,40_ = 0.49, *p* = 0.74) nor was there any significant day × treatment interaction (responses: *F*_4,40_ = 0.06, *p* = 0.99; water: *F*_4,40_ = 1.37, *p* = 0.26). There was not a significant attenuation of the effect of CPD 1 over 5 days (*F*_4,20_ = 1.46, *p* = 0.25). Data are means ± SEM of 5 rats/group.

Schedule-induced polydipsia was likewise studied over a 5-day dosing period. Although there was a tendency for the effects of CPD 1 (10 mg/kg) to abate over days, these effects were not significant (Figure 10). CPD 1 (10 mg/kg) significantly decreased excessive water consumption (*F*_1,40_ = 128.5, *p* < 0.0001) and to a lesser extent response output over 5 days (*F*_1,40_ = 11.7, *p* < 0.05). The factor of day of dosing did not significantly impact effects of CPD1 (responses: *F*_4,40_ = 0.11, *p* = 0.98; water: *F*_4,40_ = 0.49, *p* = 0.74) nor was there any significant day × treatment interaction (responses: *F*_4,40_ = 0.06, *p* = 0.99; water: *F*_4,40_ = 1.37, *p* = 0.26). There was not a significant attenuation of the effect of CPD 1 over 5 days (*F*_4,20_ = 1.46, *p* = 0.25).

Rotarod performances of mice were also studied. On day 1, vehicle produced falling in 3/12 mice and failures in 1/12; CPD 1 (10 mg/kg) did not significantly differ compared to vehicle (2/12 falls and 0/12 failures). On the fifth day of dosing, vehicle produced falls in 1/12 mice and failures in 0/12. CPD 1 after 5 days of dosing produced neither falls nor failures in performance in the 12 mice tested.

## Discussion

Obsessive–compulsive disorder has been estimated to affect from 1 to 3% of the general population (1), has high comorbidity with other psychiatric disorders (4, 5), and patients with OCD are generally not fully served by current medications or psychotherapeutic interventions (6). The possibility that selective activation of 5-HT_2C_ receptors might be therapeutic (see Section “Introduction” for supporting evidence) was investigated with the use of a novel, selective 5-HT_2C_ receptor agonist in the present study. Prior work with another 5-HT_2C_ receptor agonist, Ro 60-0175, provided impetus for the current investigations (19). In the present series of experiments, a novel benzofuranylpiperazine molecule with high affinity, efficacy, and selectivity for 5-HT_2C_ receptors (CPD 1) was studied on rodent behaviors that were excessive and non-habituating in both rat and mouse models and for which SSRIs that help in some OCD patients are active.

The overall findings that CPD 1 suppressed a host of excessive and repetitive behaviors without notable effects on ancillary behaviors suggest that 5-HT_2C_ receptor agonists may be efficacious in the clinical management of OCD. Thus, in contrast to the SSRIs studied, CPD 1 while suppressing excessive drinking did not significantly alter responding controlled by food presentation of rats. Further, the behavior of the rats generally returned to baseline levels on the subsequent experimental session conducted. In mouse experiments, it was demonstrated that CPD 1 was also effective in decreasing excessive marble-burying and nestlet shredding at doses lower than those that suppressed other behaviors (rotarod or locomotion).

The affinity of CPD 1 across serotonin receptors (h5-HT_1–7_) was assessed using standard radioligand-binding techniques employing agonist radioligands. CPD1 exhibited high affinity for the h5-HT_2C_ receptor; CPD 1 was 22-fold selective over h5-HT_2A_ receptors, 50-fold selective over h5-HT_2B_ receptors, and at least 100-fold selective over other cloned h5-HT receptors. The efficacy of CPD 1 was shown to be a full agonist at 5-HT_2C_ receptors but a partial agonist at 5-HT_2A_ and 5-HT_2B_ receptors. CPD 1 was also selective over 68 other protein targets evaluated with affinities for only 23.5% of these that reached a level of >50% inhibition at 10 µM. With an affinity of 2 nM at 5-HT_2C_ receptors, these values give CPD 1 a selectivity of five orders of magnitude over these other proteins. Although this molecule is highly selective for h5-HT_2C_ receptors, the selectivity data were generally obtained from human recombinant systems. Additional studies in rat cortical tissues and central drug exposure studies to relate drug concentrations to receptor affinities will be needed to confirm the translation of *in vitro* selectivity to *in vivo* efficacy by virtue of.engagement with central 5-HT_2C_ receptors. The data we presented here with the selective 5-HT_2C_ and-HT_2A_ receptor antagonists are a step already in that direction.

We demonstrated here that the SSRIs fluoxetine and clomipramine decrease the excessive drinking produced by an intermittent schedule of food delivery. Schedule-induced polydipsia is excessive and non-habituating and serves no known viable biological function (16). Under conditions where standard OCD treatment agents are active (SSRIs), we showed that the non-selective 5-HT_2C_ agonist mCPP (active in OCD patients) and the selective 5-HT_2C_ agonists Ro 60-0175 and CPD 1 were also effective in decreasing excessive drinking behavior of rats generated by schedule-induction. It was also shown that CPD 1 suppressed non-habituating behaviors such as marble-burying and nestlet shredding in mice, and drinking induced by water deprivation and by isoproterenol in rats. A range of behaviors that might be characterized as OCD-like were also attenuated by Ro 60-0175 (19).

The mechanism by which effects of CPD 1 were produced *in vivo* was shown to be due to agonist activity at 5-HT_2C_ receptors. Effects on excessive drinking, marble-burying, and nestlet-shredding were markedly and significantly attenuated in the presence of the 5-HT_2C_ receptor antagonist SB242084. The antagonism was pharmacologically specific since SB242084 did not block the effects of d-amphetamine on excessive drinking. Further, a selective antagonist of 5-HT_2A_ receptors (MDL 100907) was not an effective blocker.

In contrast, the conclusion that 5-HT_2C_ receptor antagonists might be viable OCD treatments was rendered by Papakosta et al. (22) from data in a behavioral pharmacology study examining alternation behavior. However, the study did not evaluate 5-HT_2C_ receptor agonists and, therefore, no data on this mechanism can be gained from their study. Similarly, a report by Tucci et al. (23) evaluated the role of 5-HT_2A/C_ receptors in the anti-OCD-like efficacy of mCPP in attenuating quinpirole-induced compulsive checking behaviors in rats. They concluded that mCPP effects were not mediated by 5-HT_2A/C_ receptors since ritanserin did not block its effects. As in the study by Papakosta et al. (22), the data raise the possibility that effects of mCPP are not driven by 5-HT_2C_ receptors despite data that support a 5-HT_2C_ mechanism of action of mCPP (24). Nonetheless, neither study provides data to question the hypothesis of the present work and that of Martin et al. (19) that 5-HT_2C_ receptor agonists prevent OCD-like behaviors and do so as a result of interaction with 5-HT_2C_ receptors. Indeed, lorcaserin (Belviq^®^), a drug approved for control of obesity, reduced impulsive behaviors (premature responses or reducing false alarms) in rats as did another selective 5-HT_2C_ receptor agonist CP-809101 (25). The data suggested to Higgins and coauthors that the actions of lorcaserin might be to impede relapse to eating as an impulse control brake.

In contrast to the SSRIs and to mCPP and Ro 60-0175, CPD 1 was the only treatment that reduced excessive drinking with high efficacy without at the same time affecting other ongoing behaviors (e.g., lever pressing controlled by food delivery). CPD 1 also suppressed marble-burying and nestlet shredding in mice at doses without motoric impairing or stimulating effects. The differentiating pharmacology that accounts for the unique behavioral specificity of CPD 1 relative to other 5-HT_2C_ receptor agonists is not clear. Both Ro 60-0175 and CPD1 are high affinity and full agonists at 5-HT_2C_ receptors [(19); Tables 1 and 2]. Regardless of the mechanisms ultimately ascribed to this selectivity, the ability of CPD 1 to affect target behaviors with little impact on ancillary behaviors is likely a phenomenon of therapeutic and safety significance (26). Given the clinical use of lorcaserin for obesity, this molecule could be used now as a clinical test of the 5-HT_2C_/OCD hypothesis.

## Ethics Statement

The facilities in which the animals were maintained are fully accredited by the American Association for the Accreditation of Laboratory Animal Care (AAALAC), and the studies described herein were conducted in accordance with the Guide for Care and Use of Laboratory Animals under protocols approved by a local animal care and use committee. All work was done in accordance with U.S. Public Service Policy on Humane Care and Use of Laboratory Animals, amended August 2002. Furthermore, all research protocols were approved by an internal Animal Care and Use Committee of the Lilly Research Labs.

## Author Contributions

MR analyzed the data and wrote the manuscript; CO conducted experiments and wrote the manuscript; DL conceptualized the study; XL conducted experiments, analyzed the data, and wrote the manuscript; DM and RC conducted experiments; DN contributed the data and wrote the manuscript; KB contributed the research tools; JW contributed the data, analyzed the data, and wrote the manuscript.

## Conflict of Interest Statement

All authors were employees of Eli Lilly and Co. at the time of this study.

## Abbreviations

CPD 1, ((3S)-3-methyl-1-[4-(trifluoromethyl)-7-benzofuranyl]-piperazine); Mcpp, *meta*-chlorophenylpiperazine; MDL100907, (*R*)-(2,3-dimethoxyphenyl)-[1-[2-(4-fluorophenyl)ethyl]-4-piperidyl]methanol; Ro 60-0175, (S)-6-chloro-5-fluoro-1H-indole-2-propanamine; SB-242084, 6-chloro-5-methyl-*N*-{6-[(2-methylpyridin-3-yl)oxy]pyridin-3-yl}indoline-1-carboxamide.
